# Effectiveness of the BreatheSuite Device in Assessing the Technique of Metered-Dose Inhalers: Validation Study

**DOI:** 10.2196/26556

**Published:** 2021-11-03

**Authors:** Meshari F Alwashmi, Gerald Mugford, Brett Vokey, Waseem Abu-Ashour, John Hawboldt

**Affiliations:** 1 BreatheSuite Inc St. John's, NL Canada; 2 Memorial University St. John's, NL Canada

**Keywords:** digital health, asthma, smartphone, mHealth, mobile health, effective, observational, treatment, chronic obstructive pulmonary disease, inhaler, app

## Abstract

**Background:**

The majority of medications used in treating asthma and chronic obstructive pulmonary disease (COPD) are taken through metered-dose inhalers (MDIs). Studies have reported that most patients demonstrate poor inhaler technique, which has resulted in poor disease control. Digital Health applications have the potential to improve the technique and adherence of inhaled medications.

**Objective:**

This study aimed to validate the effectiveness of the BreatheSuite MDI device in assessing the technique of taking a dose via an MDI.

**Methods:**

The study was a validation study. Thirty participants who self-reported a diagnosis of asthma or COPD were recruited from community pharmacies in Newfoundland and Labrador, Canada. Participants used a BreatheSuite MDI device attached to a placebo MDI and resembled taking 3 doses. Pharmacists used a scoring sheet to evaluate the technique of using the MDI. An independent researcher compared the results of the pharmacist’s scoring sheet with the results of the BreatheSuite device.

**Results:**

This study found that the BreatheSuite MDI can objectively detect several errors in the MDI technique. The data recorded by the BreatheSuite MDI device showed that all participants performed at least one error in using the MDI. The BreatheSuite device captured approximately 40% (143/360) more errors compared to observation alone. The distribution of participants who performed errors in MDI steps as recorded by BreatheSuite compared to errors reported by observation alone were as follows: shaking before actuation, 33.3% (30/90) versus 25.5% (23/90); upright orientation of the inhaler during actuation, 66.7% (60/90) versus 18.87% (17/90); coordination (actuating after the start of inhalation), 76.6% (69/90) versus 35.5% (32/90); and duration of inspiration, 96.7% (87/90) versus 34.4% (31/90).

**Conclusions:**

The BreatheSuite MDI can objectively detect several errors in the MDI technique, which were missed by observation alone. It has the potential to enhance treatment outcomes among patients with chronic lung diseases.

## Introduction

Many medications used in treating asthma and chronic obstructive pulmonary disease (COPD) are administered via inhalation devices. They come in various forms, but the most common form is metered-dose inhalers (MDIs) [1-3]. These inhalers include salbutamol, fluticasone, and ciclesonide. These inhalers are primarily used to manage symptoms and prevent exacerbations. Both the Global Initiative for Asthma (GINA) [4] and the Global Initiative for Chronic Obstructive Lung Disease (GOLD) [5] recommends using inhalers to achieve good symptom control, minimize future risk of exacerbations, and improve exercise tolerance.

The Aerosol Drug Management Improvement Team, which GINA supports, provided information on the proper way of using an MDI [6]. Furthermore, each drug manufacturer provides instruction on using an MDI and including it in the drug pamphlet. Nevertheless, studies have reported that up to 92% of patients with asthma demonstrate poor inhaler technique [7-9]. Sanchis et al [10] conducted a systematic review in 2016 to assess the most common errors in inhaler use among patients with asthma and those with COPD treated with MDIs. They concluded that incorrect inhaler use is unacceptably high outside clinical trials and does not seem to have improved over the past 40 years [10]. Similarly, Press et al [11] in 2011 examined the rates of inhaler misuse among patients with asthma and those with COPD; they concluded that misuse rates are prevalent among both groups. Improper inhaler technique can significantly affect the amount of medication reaching the lungs, leading to poor symptom control and more emergency department visits [12,13]. Errors in inhaler technique and nonadherence can affect medication delivery and decrease the benefits of taking the medication [14]. A systematic review of errors in the inhaler technique suggests that most reported errors were in coordination, speed of the inhalation, depth of inspiration, and no postinhalation breath-holding [10]. To improve the inhaler technique, researchers recommend frequent assessment of inhalation technique [15,16]. One of the literature’s current gaps concerns the most appropriate method to intervene if patients continue to misuse their inhalers [16]. Innovative technologies have been introduced, which may improve inhaler technique and consequently improve health outcomes [17-19].

Digital Health interventions have been increasing in the past 10 years, with significant advances in mobile apps, web portals, and electronic inhaler sensors. Researchers are now proposing digital health applications for many complex health conditions, including asthma and COPD. These technologies allow patients and health care providers to monitor and manage their symptoms more effectively. Several studies demonstrated improved clinical outcomes from implementing these technologies [17,19-21]. An example includes a meta-analysis that determined electronic reminders can improve patient adherence to inhaled corticosteroids by 19% [22]. One advantage of using digital health applications is having long-term data collection of symptoms, triggers, and inhaler use, which permits the identification of necessary changes to assist patients and their caretakers in understanding if symptoms are exacerbating [23,24].

The BreatheSuite MDI device is an auxiliary, add-on device which is connected to an approved MDI. It passively and quantitatively monitors important inhaler adherence and technique metrics, providing user feedback through a linked mobile app. This information may be used by patients to improve inhaler technique, and may also be shared with health care providers. There are several devices that monitor the adherence of MDIs [25], but there is a paucity in MDIs that monitor both adherence and technique. This study aims to validate the effectiveness of the BreatheSuite MDI device in assessing the technique of taking a dose via an MDI.

## Methods

### Purpose

This study aimed to validate the effectiveness of the BreatheSuite MDI device in assessing the technique of taking a dose via an MDI. 

### The BreatheSuite MDI

**Figure 1:**
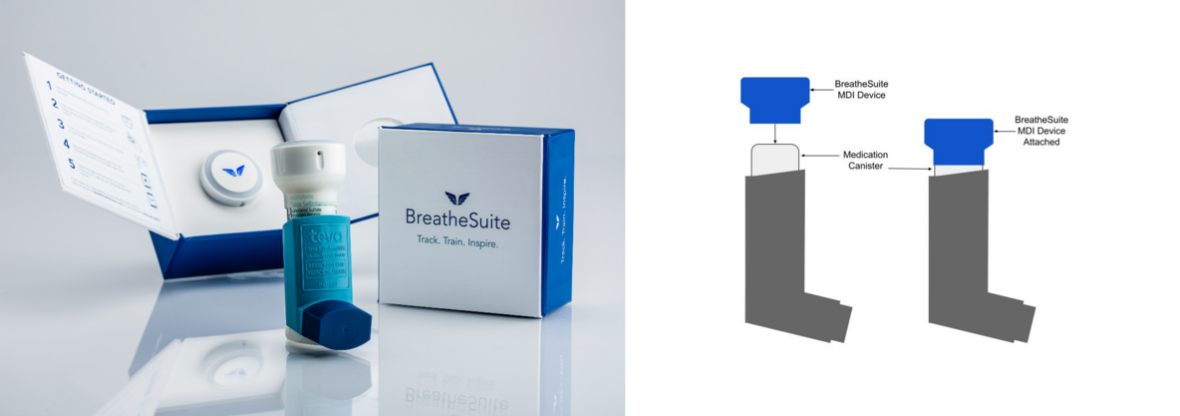
Placebo inhaler with the BreatheSuite metered-dose inhaler device.

The BreatheSuite MDI device is an auxiliary, add-on device which is connected to an approved MDI. It is mounted by placing the device over the canister of a standard inhaler. The inhaler is used in the same way that it would be used without the BreatheSuite MDI Device. The device is designed for passive monitoring; it ensures that patients can continue to follow their prescriptions. The BreatheSuite MDI device is approximately 1 inch in diameter and attaches to a standard MDI canister with an elastic sleeve, as shown in the image below (Figure 1).

The BreatheSuite MDI device has the potential to improve the technique and adherence of inhaled medications. It uses quantitative measures to assess several inhaler technique metrics. These inhaler technique metrics are based on the inhaler manufacturer instructions as well as quantitative analysis performed on lung deposition as a result of impacts of several inhaler technique parameters. This has resulted in the quantitative analysis of the following:

1. Shaking duration (if the inhaler was shaken for 3 seconds): The duration of shaking was measured to within 0.1 seconds and rounded to the nearest 0.5 seconds; thus, any duration greater than 2.75 seconds would be considered acceptable.
2. Orientation (whether the device was oriented with the mouthpiece straight toward the back of the throat): The BreatheSuite device measures orientation from +1° to –1° and the acceptable range is from –10° to +20°, with 0° being the location where the mouthpiece is horizontal and points toward the back of the throat.
3. Press timing (if the user began inhaling before actuating the inhaler canister): The press timing is defined as the difference between the actuation time and the inhalation start time (each measured to within +0.1 and –0.1 seconds). The resultant value must be between –1.0 and +1.0 to be considered acceptable.
4. Inhalation duration (if the user has inhaled for at least 3 seconds): This value is defined as the difference between the inhalation end time and the inhalation start time, or actuation time, whichever is shortest. This value is then rounded to the nearest 0.5. Any duration above 3 seconds is considered acceptable.

This information is then transmitted to the BreatheSuite mobile app via Bluetooth and is securely uploaded to a remote server. The user will receive technique-related feedback and subsequent correcting advice by accessing the mobile app. The data are transferred from the app to a secure cloud database such that it is available for analysis and review. Health care providers can view the data to determine if additional training is necessary to improve inhaler technique.

### Study Design

The study was a validation study.

### Recruitment and Study Setting

We recruited patients from 2 community pharmacies in the province of Newfoundland and Labrador, Canada. One pharmacy was located in an urban area, while the other pharmacy was in a rural area. One pharmacist at each pharmacy was responsible for recruitment and data collection.

A convenience sample of 30 patients was recruited during their routine visits to the pharmacy. The pharmacist invited patients to participate in the study if they met the following eligibility criteria: being able to communicate in English, having a self-reported diagnosis of asthma or COPD, being 18 years or older, and having been prescribed an MDI and having used it in the past. First-time users of MDI were excluded.

### Ethical Considerations

Ethical approval for this study was obtained from the Newfoundland and Labrador Health Research Ethics Authority. Before agreeing to participate, all subjects were informed about the nature of the study, potential risks and benefits, and their rights as research subjects. All participants completed a written consent form. They were also given a copy of the consent form. Each participant and pharmacist were offered a gift card to compensate them for their participation time.

### Data Collection

Before enrolling participants, all pharmacists were trained to follow the study protocol, including participant recruitment, baseline questionnaire, and scoring sheet. Each participant completed a baseline study questionnaire about their demographics and smartphone use (Multimedia Appendix 1). After completing the questionnaire, the pharmacist gave participants a BreatheSuite MDI device attached to a placebo MDI (Figure 1). Participants did not receive instructions on how to use the device. They were asked to take 3 doses in the same manner as they would use their inhalers. The BreatheSuite MDI device evaluated the following technique parameters:

1. Was the MDI was properly shaken (at least for 3 seconds)?
2. Was the MDI in the upright position before taking the dose?
3. Was the MDI actuated after starting to take a breath?
4. Was the duration of the inhalation more than 3 seconds?

Pharmacists then used a scoring sheet to evaluate the technique of the participant using the MDI. The scoring sheet followed the same parameters that were evaluated by the BreatheSuite MDI (Multimedia Appendix 2). The pharmacists evaluated the technique parameters by using binary answers (yes/no). After the scoring was complete, pharmacists trained participants on how to use inhalers correctly. Each patient and pharmacist and patient will be offered a gift card of Can $20 (US $16.15) to compensate for their participation time.

### Blinding

The pharmacist was blinded to the results from the BreatheSuite device. An independent researcher compared the results of the pharmacist’s scoring sheet with the results of the BreatheSuite device.

### Statistical Analysis

A database of the questionnaire results was created using unique nonidentifying numbers. The information was password-protected. Before conducting the analysis, data were cleaned, coded, and entered into SPSS (version 25.0, IBM Corp). Unclear or incomplete survey items were flagged for queries. These were brought to the attention of the research team, each item was discussed, and a decision concerning its eligibility and entry was made. Baseline characteristics of participants were summarized with percentages for categorical variables and mean (SD) values for continuous variables. We reported the frequencies and percentages of technique errors from direct observations and the BreatheSuite MDI. Additionally, we calculated Cohen *κ* values to determine if there was agreement between the pharmacist’s observations and the BreatheSuite device.

An independent research analyst, who was not involved in recruiting and training the pharmacists, analyzed the results of the pharmacist scoring sheet with the scoring from the BreatheSuite MDI device. The analyst was able to link both data sets using the time of taking the dose. The pharmacist recorded the time, including seconds, in the scoring sheet. The time was also stored in the database automatically.

## Results

### Participant Characteristics

A total of 30 patients participated in the study. Approximately 60% (18/30) of participants were male. The mean age of participants was 56.5 (range 33-73) years. The highest level of education for most participants was a high school degree 46.6% (14/30). Half of the participants were living in a rural area 50% (15/30). Although more than half of the participants use mobile apps, only 16.6% (5/30) use health apps. Almost 80% (24/30) of participants did not use a spacer or aerochamber. Table 1 illustrates the participant characteristics.

**Table 1:** Participant characteristics (N=30).

Variables			Values
Age (years), mean (SD)			56.6 (10.5)
**Gender, n (%)**			
	Males	18 (60)	
	Females	12 (40)	
**Education, n (%)**			
	General education diploma	3 (10)	
	High school	14 (46.6)	
	Bachelors	0 (0)	
	Masters	1 (3.3)	
	PhD/MD/JD	0 (0)	
	Other	12 (39.9)	
**Locality of residence, n (%)**			
	Rural	15 (50)	
	Small	3 (10)	
	Medium	2 (6.6)	
	Large	9 (30)	
	other	1 (3.3)	
**App use, n (%)**			
	No	13 (43.3)	
	Yes	16 (53.3)	
	Do not know	1 (3.3)	
**Health app usage, n (%)**			
	No	23 (76.6)	
	Yes	5 (16.6)	
	Do not know	2 (6.6)	
**Spacer/aerochamber use, n (%)**			
	No	21 (70)	
	Yes	6 (20)	
	Do not know	1 (3.3)	
	I was instructed to use it but I do not use it	2 (6.6)	

### Comparison of Subjective and Objective Measures of Inhaler Technique

**Figure 2:**
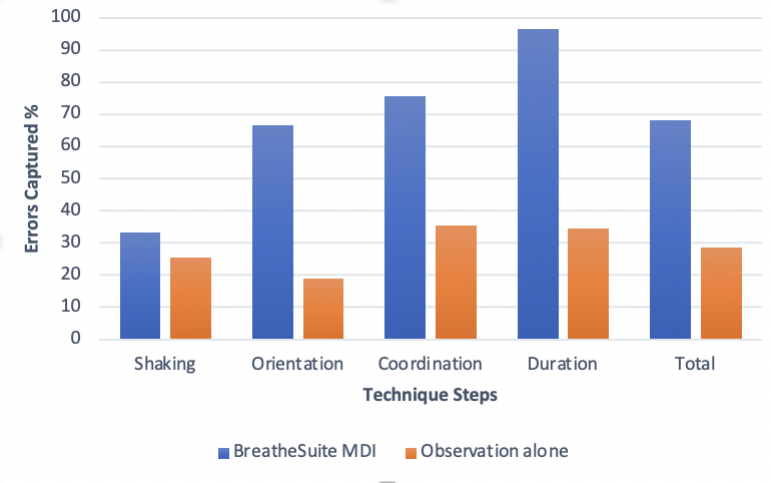
The percentage of captured errors in MDI steps recorded by BreathSuite compared to errors reported by observation alone.

Each participant simulated taking 3 doses using the placebo MDI with the BreatheSuite MDI device, resulting in 90 doses for analysis. For each dose, we measured 4 technique metrics, which resulted in 360 measurements. The data recorded by the BreatheSuite MDI device showed that all participants performed at least 1 error in using the MDI. Among the metrics collected by both the pharmacist and the BreatheSuite MDI, the BreatheSuite MDI device captured 68.3% (246/360) of the errors made by the participant compared to 28.6% (103/360) errors captured by observation alone (Figure 2). The subjective and objective measures of inhaler technique included are shakes, orientation, coordination, and duration. The distribution of technique errors in MDI steps recorded by the BreatheSuite MDI compared to errors reported by observation alone were as follows: shaking before actuation, 33.3% (30/90) versus 25.5% (23/90); upright orientation of inhaler during actuation, 66.7% (60/90) versus 18.87% (17/90); coordination (actuating after the start of inhalation), 76.6% (69/90) versus 35.5% (32/90); duration of inspiration, 96.7% (87/90) versus 34.4% (31/90). Figure 2 highlights the percentage of errors in MDI steps recorded by the BreatheSuite MDI compared to errors reported through observation alone. The following are the Cohen *κ* values: shaking (fair agreement, *κ*= 0.283), orientation (slight agreement, *κ*= 0.183), coordination (slight agreement, *κ*= 0.071), and duration (no agreement, *κ*=–0.03).

## Discussion

### Principal Findings

This study has indicated that the BreatheSuite MDI can objectively detect MDI technique errors, and in some cases can be better at assessing various quantitative metrics that are difficult to assess through observation alone. For example, measuring minor inhaler orientation angle deviations by objective observation can be difficult, along with minor differences in inhalation duration. In total, the BreatheSuite MDI device captured approximately 40% (143/360) more errors compared to observation alone.

The low Cohen *κ* values and the objective nature of the BreatheSuite device potentially indicate that the BreatheSuite MDI performs better than observation alone. Objective measures of inhaler technique have the potential to become the new gold standard. Additional research is necessary to assess the relationship between objective improvements in inhaler technique with clinically significant improvements in health outcomes. 

### Comparison With Previous Work

Using technology to enhance inhaled medication plays a vital role in the management of chronic lung diseases. Several studies have assessed the effectiveness of electronic devices to an MDI to improve medication adherence [17-19]. However, there is a lack of studies and devices that assess the technique of using MDIs.

To further understand how patients use their inhalers, some pharmaceutical companies started manufacturing digital inhalers, such as the Digihaler. The Digihaler captures the inhalation rate when taking a dose, as well as opening and closing the inhaler cap. Studies conducted on Digihaler concluded that it could capture objective technique data [26]. These data may help identify clinically meaningful information early and facilitate physician-patient interventions and conversations [27]. It is important to consider the cost of these digital inhalers, especially when considering the usability and interoperability of using several digital inhalers from different drug manufacturers.

Some of our findings confirm those previously reported in the context of using electronic MDI devices to assess MDI technique. Our findings are in agreement with those of Biswas et al [28], who used an objective measure for inhaler technique and demonstrated that 100% of the patients made at least 1 error in using an MDI. As Biswas et al [28] noted, data recorded by an objective MDI device provides accurate measurements of MDI use, which could help evaluate how effectively patients use their MDIs. The major difference between the device used by Biswas et al [28] and the BreatheSuite device is that the latter does not require charging; the battery in the BreatheSuite device can operate for more than a year without charging [28]. This sustained battery life promotes usability through passive data collection.

### Strengths and Limitations

The pharmacists were blinded to the BreatheSuite MDI data. An independent analyst compared the data between the BreatheSuite MDI and the pharmacist scoring sheet. Digital applications may be important in geographic locations with relatively large numbers of rural residents, such as Newfoundland and Labrador. Digital applications may enhance care provider access throughout sparsely populated rural areas as they can access information remotely. Half of the study sample was from a rural area, which supports the generalizability of our findings to rural areas.

There were also several limitations of note. Although the sample may not be generalizable to all patients with asthma and those with COPD; the study had broad inclusion criteria to resemble the target population. The BreatheSuite MDI does not track all the steps required to use an inhaler, such as exhaling before taking a dose and holding one’s breath after inhalation. However, it takes the majority of the technique steps that have the potential to improve inhaler technique and supplement the teaching offered by health care professionals.

### Implications for Practice and Future Research

This study provides insights into the effectiveness of the BreatheSuite MDI drive in capturing errors in MDI use. This information may help a variety of stakeholders (eg, health care providers, patients, administrators, and technology developers) who are planning to use an objective measure of MDI adherence and technique. The BreatheSuite MDI will transmit this information to the BreatheSuite mobile app. Patients can then receive technique-correcting advice by accessing the mobile app. In addition, health care providers can view the data to determine if additional training is necessary to improve the technique or adherence to using MDIs. These data can also be used to identify clinically meaningful information early such as (eg, rescue to controller usage ratios) and facilitate meaningful physician-patient conversations.

Digital applications that assess inhaled medications are increasingly gaining importance in managing chronic lung diseases [17-19]. These applications have the potential to improve health outcomes while reducing health care costs.

Future studies should examine the sustainability of behavior change following the use of the BreatheSuite MDI device. They should also include an experimental design to assess the BreatheSuite MDI’s effectiveness in improving clinical outcomes among patients diagnosed with chronic lung diseases. A larger and more heterogeneous sample with a longer follow-up period could confirm the study findings and expand the knowledge around the effectiveness of devices such as the BreatheSuite MDI.

### Conclusions

Our findings potentially indicate that the BreatheSuite MDI can objectively detect several MDI technique errors missed by observation alone. The BreatheSuite MDI has the potential to enhance treatment and therefore improve outcomes among patients with chronic lung diseases. Additional studies are required to examine the effectiveness of the BreatheSuite MDI device on clinical outcomes.

## Appendix

Multimedia Appendix 1 Baseline Questionnaire.

Multimedia Appendix 2 Pharmacist Scoring Sheet.

## Abbreviations

COPD: chronic obstructive pulmonary disease
GINA: Global Initiative for Asthma
GOLD: Global Initiative for Chronic Obstructive Lung Disease
MDI: metered-dose inhaler
