# Supplementary material for: Effectiveness of the BreatheSuite Device in Assessing the Technique of Metered-Dose Inhalers: Validation Study
Source: JMIR Biomed Eng. 2021 Nov 3;6(4):e26556. doi: 10.2196/26556 (PMC11041462; doi:10.2196/26556)
Supplement: Multimedia Appendix 1 [file biomedeng_v6i4e26556_app1.pdf]

Appendix 1: Baseline Questionnaire

Name:

Study ID:

1. How old are you?

____

2. What is your sex?

O Female

O Male

3. To which gender identity do you most identify?

O Female

O Male

O Prefer not to answer

O Other

If other , please specify:_________________

4. What is your highest level of education?

O High School Equivalency (GED)

O High School

O Bachelor’s degree

O Master’s degree

O PhD/MD/JD

O Prefer not to answer

O Other

If other, please specify:____________________________________

5. Which of the following best describes the area you currently live in?

O Rural area, with a population less than 1,000

O Small population centre, with a population between 1,000 and 29,999

O Medium population centre, with a population between 30,000 and 99,999

O Large urban population centre, with a population of 100,000 or more.

6. Do you use apps on your mobile phone?

O Yes

O No

O I don’t know

7. Do you use health related apps on your mobile phone?

O Yes

O No

O I don’t know

8. Do you use a spacer or an aerochamber with your inhaler?

O Yes

O No

- O I was instructed to use it but I don’t use it

- O I don’t know

9. Would you be interested in participating in an interview to discuss the BreatheSuite app and device?

- O Yes

- O No

10. Are you willing to be contacted for future research with BreatheSuite?

- O Yes

- O No

11. If you answered yes to questions 9 or 10, please provide a phone number and/or email below to allow us to contact you.

Name:

Phone number:

Email:

Best time to call:

Thank you for participating in this study, your answers to these questions are very important to us , and we really appreciate you taking the time to complete this questionnaire.
