# Supplementary material for: Effectiveness of the BreatheSuite Device in Assessing the Technique of Metered-Dose Inhalers: Validation Study
Source: JMIR Biomed Eng. 2021 Nov 3;6(4):e26556. doi: 10.2196/26556 (PMC11041462; doi:10.2196/26556)
Supplement: Multimedia Appendix 2 [file biomedeng_v6i4e26556_app2.pdf]

Appendix 2: Pharmacist Scoring Sheet

Participant Study ID:

Time of starting the test: __________

Technique	Dose 1	Dose 2	Dose 3
the MDI was properly shaken (at least for 3 seconds)			
the MDI is in the UPRIGHT position before taking the dose			
The MDI was actuated after starting to take a breath			
the duration of the inhalation was more than 3 seconds			

- Please answer using Yes or No

Time of completing the test: __________

Notes:
